# Herausforderung Abstinenz – Fallbericht und Übersicht zur Therapie von Alkoholabhängigkeit bei Schwangerschaft

**DOI:** 10.1007/s40211-020-00367-5

**Published:** 2020-10-29

**Authors:** Stephan Listabarth, Daniel König, Andreas Wippel, Nathalie Pruckner, Deirdre Maria Castillo, Sandra Vyssoki, Andrea Gmeiner

**Affiliations:** 1 Klinische Abteilung für Sozialpsychiatrie, Universitätsklinik für Psychiatrie und Psychotherapie, Medizinische Universität Wien, Währinger Gürtel 18–20, Wien, 1090 Österreich; 2 Universitätsklinik für Gynäkologie und Geburtshilfe, Medizinische Universität Wien, Wien, Österreich; 3 Department Gesundheit, FH St. Pölten, St. Pölten, Österreich

**Keywords:** Fetales Alkoholsyndrom, Schwangerschaft, Alkoholismus, Rückfallsprophylaxe, Fetal alcohol syndrome, Pregnancy, Alcoholism, Relapse prevention

## Abstract

Die potenziell teratogenen Effekte von Alkoholkonsum in der Schwangerschaft stellen wichtige medizinische sowie sozio-ökonomische Faktoren dar. Die Einhaltung einer strikten Alkoholkarenz während der Schwangerschaft, stellt allerdings besonders für Frauen, welche an einer Alkoholabhängigkeit leiden, eine große Herausforderung dar. Eine optimale psychopharmakologische Behandlung der werdenden Mütter ist auf Grund unzureichender Studienlage oder bekannter Teratogenität selten möglich. Die aktuell zugelassenen Substanzen zur Anticraving- sowie Rückfallsprophylaxe bei Alkoholabhängigkeit sind in der Schwangerschaft kontraindiziert. Die off-label Verwendung von Ondansetron (5-HT_3_-Rezeptor-Antagonist) als Rückfallsprophylaxe ist eine mögliche therapeutische Option. Im hier dargestellten Fall erhielt eine schwangere Patientin eine psychopharmakologische Behandlung mit Sertralin, Quetiapin sowie Ondansetron. Unter dieser Therapie konnte während der Schwangerschaft eine durchgehende Abstinenz bei der Patientin erreicht werden.

## Summary

The potentially deleterious and teratogen effects of alcohol consumption during pregnancy are an important medical as well as socio-economic topic. Maintaining the necessary strict abstinence is especially challenging for pregnant women diagnosed with alcohol dependence. Due to limited data, optimal pharmacological treatment is seldom possible. Importantly, all substances currently approved for relapse prevention are contraindicated during pregnancy. Limited data concerning the off-label-use of Ondansetron (5-HT_3_ receptor antagonist) as relapse prevention during pregnancy exists. In the portrayed case-report, a pregnant patient received psychopharmacological treatment with Sertraline, Quetiapine and Ondansetron. Under this established pharmacological therapy, the patient achieved abstinence for the remainder of the pregnancy.

## Hintergrund

Alkoholkonsum und dessen potenziell teratogener Effekt während der Schwangerschaft stellt einen wichtigen medizinischen sowie sozio-ökonomischen Faktor in der Behandlung von alkoholabhängigen Müttern dar: Für Alkoholkonsum während der Schwangerschaft sind signifikant erhöhte Raten von Spontanaborten [1], Totgeburten [2] sowie Frühgeburtlichkeit beschrieben [3]. Weiters sind, zum Teil schwerwiegende, Folgeerscheinungen eines mütterlichen Alkoholkonsums in der Schwangerschaft bekannt, welche als „Fetal Alcohol Spectrum Disorders“ (FASD) zusammengefasst werden, wobei das Vollbild als „Fetal Alcohol Syndrom“ (FAS) bezeichnet wird. FASD umfassen eine Vielfalt psychiatrisch-neurokognitiver Entwicklungsstörungen, physischer Anomalien sowie Verhaltensauffälligkeiten [4]. Bereits im Jahr 1968 wurden hierzu von *Lemoine et al.* [5] Daten publiziert und 1973 wurde die klinische Manifestation von *Jones et al. *[6] wie folgt beschrieben: Defizite im pränatalen und postnatalen Wachstum, Microzephalien, kleine Lidspalten, Epikanthi, schmale Kiefer, verflachte Philtren sowie Gelenksanomalien.

Ergebnisse eines systematischen Reviews aus dem Jahr 2017 [7] zeigen, dass weltweit etwa 10 % der Frauen in der Allgemeinbevölkerung Alkohol während der Schwangerschaft konsumieren, in Europa trinken etwa 25 % der Frauen während der Schwangerschaft Alkohol. Schätzungen zu Folge gebärt eine von 67 dieser Frauen ein Kind mit FASD [7]. Bei Frauen, welche die Kriterien des „heavy drinkings“ (laut National Institute On Alcohol Abuse And Alcoholism [8] bei Frauen mehr als drei alkoholische Getränke pro Tag) erfüllen, steigt, den Schätzungen zu Folge, die Zahl der Kinder mit FASD auf eine von 23 Geburten [7]. Die weltweite Prävalenz von FASD wird auf etwa 15 von 10.000 Personen geschätzt [7]. Es ist zu vermuten, dass bei einer großen Anzahl von Fällen die Diagnose, beeinflusst vom sozialen Stigma, der Komplexität der Diagnostik und der unspezifischen klinischen Symptomatik gar nicht gestellt wird [9]. Zusätzlich erschwert die Tatsache, dass einige Symptome nur gering ausgeprägt sind oder sich teilweise erst mit einer gewissen Latenz manifestieren, die Diagnosestellung dieser Erkrankung.

Eine der umfassendsten Guidelines zur Diagnostik des FAS und der damit verbundenen Erkrankungen, wurde im Jahr 2005 in Kanada erarbeitet [10]. Darin wird empfohlen den diagnostischen Prozess in sechs Unterpunkte zu teilen: (1) Screening und Zuweisung, (2) Physikalische Untersuchung und Differentialdiagnostik, (3) Erfassung neurologischer Verhaltensweisen, (4) Behandlung und Follow-Up, (5) Mütterlicher Alkoholkonsum in der Schwangerschaft, (6) Diagnostische Kriterien für FASD, FAS sowie Alkoholassoziierte Neuroentwicklungsstörung (ARND). Für den letzten Schritt des diagnostischen Prozesses wurde die „4-Digit Diagnostic Code Criteria“ entwickelt (siehe Tab. 1), wobei ein Zahlencode von 4‑4-4‑4 auf das Vorliegen eines Vollbildes eines FAS hinweist.

**Table 1:** 

	Wachstumsdefizit	FAS Gesichts Phänotyp	ZNS Schädigung oder Fehlfunktion	Pränatale Alkoholexposition
1	Nein	Nein	Nein	Kein Risiko
2	Ja, mild	Ja, mild	Möglich	Unwissenheit
3	Ja, moderat	Ja, moderat	Wahrscheinlich	Etwas Risiko
4	Ja, signifikant	Ja, signifikant	Definitiv	Hohes Risiko

Die Datenlage zum Erwachsenenleben mit diagnostizierter FASD ist bisher äußerst limitiert. *Streissguth et al.* [11] führten in den 90er Jahren eine Follow-Up-Studie bei erwachsenen PatientInnen (Mittleres Alter: etwa 25 Jahre) mit in der Kindheit diagnostizierter FASD durch. Hierbei konnte gezeigt werden, dass 94 % der untersuchten Personen unter psychischen Problemen litten – am häufigsten wurden Aufmerksamkeitsdefizit-Hyperaktivitätsstörungen sowie Depressive Störungen genannt [11]. Weiters benötigten 80 % der untersuchten PatientInnen Unterstützung im Sinne von betreuten Wohneinrichtungen oder hatten Probleme hinsichtlich Erwerbstätigkeit [11]. Eine rezentere Studie gibt eine im Vergleich zur Gesamtpopulation deutlich reduzierte mittlere Lebenserwartung von etwa 34 Jahren für FASD-Betroffene an, wobei die Reduktion der Lebenserwartung hauptsächlich auf Suizide, Unfälle und Intoxikationen zurückzuführen ist [12].

Auf Grund von mannigfaltigen bio-psycho-sozialen Folgeerscheinungen und Belastungen ist eine strikte Alkoholkarenz in der Schwangerschaft dringend indiziert. Eine besondere Herausforderung stellt diese jedoch für Frauen dar, welche an einer Alkoholabhängigkeit leiden – unter anderem auf Grund limitierter pharmakologischer Optionen während der Schwangerschaft: Im Allgemeinen sind mit Naltrexon, Disulfiram und Acamprosat durch die Food and Drug Administration (FDA) 3 Substanzen als sogenannte Rückfallsprophylaxe bei einer bestehenden Alkoholabhängigkeit zugelassen [13], in Österreich sind Naltrexon und Disulfiram zugelassen [14]. In einigen europäischen Ländern ist auch Nalmefene [15] zugelassen. Die genannten, explizit zugelassenen Substanzen, gelten allesamt in der Schwangerschaft auf Grund des teratogenen Risikos als kontraindiziert. Als off-label Behandlungsstrategie werden unter anderem Gabapentin, Ondansetron, Topiramat und Vareniclin eingesetzt [16], die Evidenz für einen Einsatz dieser Substanzen während der Schwangerschaft ist in Tab. 2 zusammengefasst. Auch hinsichtlich einer adäquaten unterstützenden psychopharmakologischen Medikation zur Behandlung etwaiger Komorbiditäten sind die Möglichkeiten in dieser Phase deutlich eingeschränkt. Lange bestehende und eventuell suffiziente Therapieschemata müssen gegebenenfalls adaptiert werden und können so zu einer Destabilisierung oder Aggravation der Symptomatik führen. Im Folgenden wird ein Fall zu dieser Thematik aufgezeigt.

**Table 2:** 

Substanzen	Einsatz während der Schwangerschaft
Gabapentin	Eine sehr limitierte Studienlage weist auf kein speziell erhöhtes Risiko für kongenitale Fehlbildungen und andere unerwünschte Nebenwirkungen hin, allerding gibt es keine Studien die potenzielle neurologische Langzeitfolgen untersuchen [17]
Vareniclin	Auf Grund fehlender Studien ist der Einsatz während der Schwangerschaft nicht empfohlen [18]
Ondansetron	Wird mit der Indikation Hyperemesis Gravidarum während der Schwangerschaft eingesetzt. Die Datenlage zur sicheren Anwendung in der Schwangerschaft ist gut und auch hinsichtlich der Wirksamkeit als Rückfallsprophylaxe erwies sich Ondansetron als hilfreich [19, 20]
Topiramat	Auch wenn groß-angelegte Studien fehlen, gibt es Hinweise auf ein erhöhtes Risiko von kongenitalen Fehlbildungen unter Topiramat [17]

## Fallbericht

Zur Vorstellung kommt der Fall einer 30-jährigen Patientin. Die Patientin gibt an, seit ihrer Jugend vermehrt Alkohol konsumiert zu haben. Etwa seit dem 27. Lebensjahr bestünde eine Alkoholabhängigkeit. Hinsichtlich dessen gibt die Patientin an, dass sie auf Grund von psychosozialen Belastungsfaktoren vermehrt Alkohol konsumiert habe. Anamnestisch sind mehrere stationäre Voraufenthalte zur Alkoholentzugstherapie zu erheben. Bei einem früheren Aufenthalt erhielt die Patientin als Anti-Craving-Substanz Naltrexon 50 mg Tagesdosis (TD) sowie, als antidepressive Therapie, Trazodon 200 mg TD. Unter dieser Medikation konnte die Patientin nach der Entlassung aus dem stationären Aufenthalt für etwa 9 Wochen eine Abstinenz erreichen. Auf Grund einer diagnostizierten Schwangerschaft habe die Patientin im weiteren Verlauf jedoch selbstständig Naltrexon abgesetzt und es sei daraufhin zu einem Alkoholrückfall gekommen. Die Patientin berichtete eine Trinkmenge von bis zu 0,75 l Wein pro Tag über einige Wochen. Daraufhin begab sich die Patientin in der 5. Schwangerschaftswoche erneut zum Alkoholentzug und zur erneuten psychopharmakologischen Einstellung in stationäre Behandlung. Bei nur milden Entzugssymptomen sowie einer negativen Anamnese für entzugsepileptische Anfälle und dem Fehlen einer erhöhten zerebralen Erregungsbereitschaft oder einer Epilepsieneigung wurde, auf Wunsch der Patientin, von einer regelmäßigen vorgeschriebenen Benzodiazepintherapie zum Entzug abgesehen. Stattdessen wurde eine Bedarfsmedikation nach Selbstbestimmungsrecht mit jeweils 15 mg Oxazepam vereinbart. Diese Bedarfsmedikation nahm die Patientin während des Aufenthaltes lediglich zweimalig in Anspruch. Die vorbestehende Therapie mit Trazodon wurde abgesetzt und stattdessen eine antidepressive Therapie mit Sertralin 50 mg TD etabliert. Auf Grund von Schlafstörungen wurde mit der Patientin eine off-label Medikation mit Quetiapin vereinbart. In Rücksprache mit der Universitätsklinik für Gynäkologie und Geburtshilfe wurde als Anti-Craving-Substanz die oftmals auch bei Hyperemesis Gravidarum verwendete Substanz Ondansetron (5-HT_3_-Rezeptorantagonist) etabliert. Die Datenlage zur Anwendung in der Schwangerschaft gilt als gut, auch hinsichtlich Wirksamkeit als Rückfallsprophylaxe erwies sich Ondansetron als hilfreich [19, 20]. Die Entlassung erfolgte nach psychopathologischer Stabilisierung mit Sertralin 50 mg, Quetiapin 25 mg, Femibion und Ondansetron 2 mg in der 7. SSW. Bei Verlaufskontrollen zeigte sich die Patientin unter regelmäßiger Einnahme der Medikation in stabilem Zustandsbild und negierte glaubhaft jeglichen Alkoholkonsum, weshalb auf eine laborchemische Bestätigung der Abstinenz verzichtet wurde. Die Patientin blieb während der verbleibenden Schwangerschaft abstinent und brachte ein gesundes Kind zur Welt.

## Diskussion

Der dargelegte Fall beschreibt Probleme denen schwangere Frauen mit vorbestehender Alkoholabhängigkeit ausgesetzt sind. Das Erreichen des primären Ziels der absoluten Alkoholabstinenz wird für diese zu einer besonderen Herausforderung, die intensive und entsprechend sorgfältige geplante Therapieansätze benötigt.

Die wenigen vorliegenden Daten zeigen, dass Erwachsene mit diagnostizierten FASD unter diversen Langzeitfolgen leiden und, dass, Schätzungen zu Folge, etwa 15 von 10.000 Personen an FASD leiden. In Österreich gibt es, wie in den meisten anderen Ländern weltweit, keine vorliegenden Daten zur Prävalenz von FASD. Es ist jedoch anzunehmen, dass es eine hohe Dunkelziffer an Fällen gibt. Daraus ergibt sich die besondere Dringlichkeit epidemiologische, diagnostische, therapeutische aber vor allem auch präventive Aspekte dieser Erkrankung zum Gegenstand zukünftiger Forschungsprojekte zu machen. Hinsichtlich der hohen Anzahl an Frauen, insbesondere in Europa, welche in der Schwangerschaft Alkohol konsumieren, erscheint es den Autoren und Autorinnen auch als überlegenswert, Kampagnen zu forcieren, welche explizit auf die Risiken und Gefahren von Alkoholkonsum in der Schwangerschaft Hinweise geben.
